# Double ventricular response in dual AV nodal pathways mimicking interpolated premature beat

**DOI:** 10.1007/s00059-017-4539-4

**Published:** 2017-02-22

**Authors:** J.-X. Li, Q. Chen, J.-X. Hu, J.-H. Yu, P. Li, K. Hong, Q.-H. Wu, Y.-Q. Wu, X.-S. Cheng

**Affiliations:** Department of Cardiovascular Medicine, The Second Affiliated Hospital of Nanchang University, Nanchang, China

**Keywords:** Tachyarrhythmia, Premature beats, Electrocardiogram, Catheter ablation, radiofrequency, Case study, Tachyarrhythmie, Extrasystolen, Elektrokardiogramm, Katheterablation, Radiofrequenz-, Fallstudie

## Abstract

Double ventricular response in dual atrioventricular (AV) nodal pathways can result in nonreentrant supraventricular tachycardia. Since this condition was first described in 1979, around 20 cases have been reported. Here, we present the case of a patient with a confirmed diagnosis of double ventricular response in dual AV nodal pathways resembling an interpolated premature beat who underwent successful radiofrequency ablation of the slow pathway.

## Zusammenfassung

Eine doppelte ventrikuläre Antwort bei dualen AV-Knoten-Leitungsbahnen kann zu supraventrikulärer Nicht-Reentry-Tachykardie führen. Seit der Erstbeschreibung dieser Erkrankung 1979 sind 20 Fälle dokumentiert worden. In der vorliegenden Arbeit wird ein Fall mit bestätigter Diagnose einer doppelten ventrikulären Antwort bei dualer AV-Knoten Leitungsbahn – einer interpolierten Extrasystole ähnelnd – dargestellt, bei dem erfolgreich eine Radiofrequenzablation der langsamen Leitungsbahn durchgeführt wurde.

Double ventricular response in dual atrioventricular (AV) nodal pathways can result in nonreentrant supraventricular tachycardia. Since this condition was first described by Csapo et al. in 1979 [4], more than 20 cases have been reported. To date, there are no reports of a double ventricular response in dual AV nodal pathways that resemble an interpolated premature beat. In such cases, misdiagnosis can easily ensue. In this article, we report the case of a patient with a confirmed diagnosis of double ventricular response in dual AV nodal pathways resembling an interpolated premature beat who underwent successful radiofrequency ablation of the slow pathway.

## Case report

**Fig. 1:**
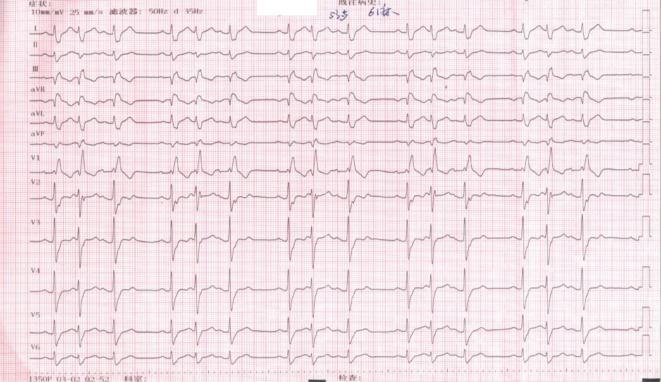
Interpolated premature beats suggesting “trigeminy.” Configuration of premature beats are essentially identical to that of sinus rhythm

**Fig. 2:**
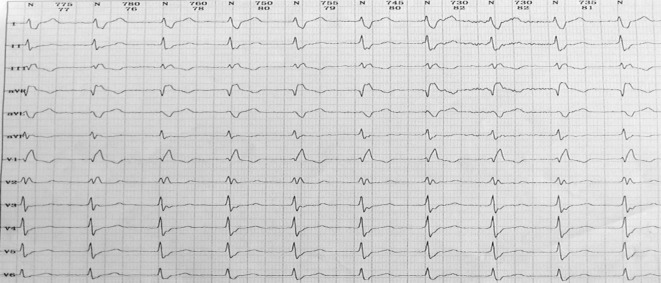
Holter ECG showing accelerated idioventricular rhythm

A 53-year-old man was admitted to the authors’ hospital with a 3-year history of recurrent palpitations and a 1-month history of chest pain. The patient had been diagnosed with interpolated premature contractions, which were refractory to metoprolol, propafenone, and amiodarone. At the time of presentation to our hospital, the patient reported no history of hypertension, diabetes, or chronic bronchitis. Serum biochemistry testing revealed normal electrolyte levels, thyroid function, and cardiac enzymes. A chest X‑ray was normal, while echocardiography identified a normal cardiac structure and function. The patient’s electrocardiogram (ECG) showed frequent interpolated premature beats suggestive of “trigeminy.” The starting vector and configuration of these premature beats were essentially identical to those of the normal sinus rhythm (Fig. 1). Holter ECG findings showed frequent interpolated premature beats and spontaneous accelerated ventricular rhythms (Fig. 2). In total, there were 48,000 premature beats during a typical 24-h period. The differential diagnosis for the origin of these interpolated premature beats included an origin from the high ventricle below the AV junction, premature junctional contractions, or dual AV nodal pathways and ventricular double response.

## Electrophysiological study and catheter ablation

**Fig. 3:**
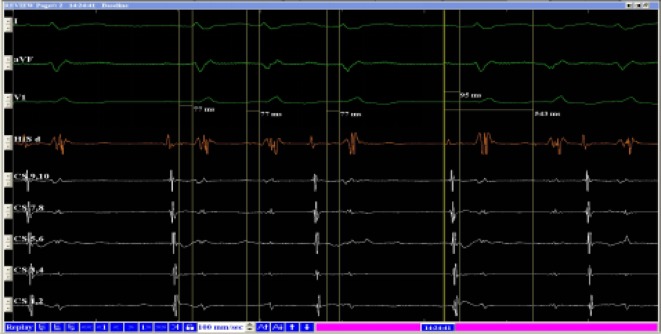
Intracardiac electrograph of premature beat

**Fig. 4:**
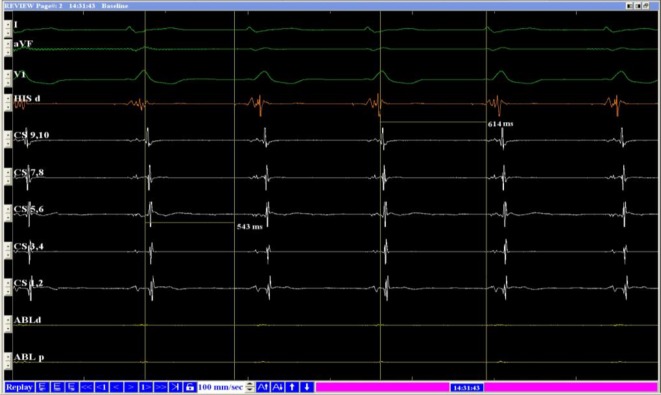
Intracardiac electrograph of ECG-accelerated idioventricular rhythm. A‑H interval is equal to A‑H conduction interval via the slow pathway shown in Fig. 3, lasting 543 ms

After obtaining informed consent from the patient, an intracardiac electrophysiological study (EPS) was subsequently performed: A 10-pole coronary venous sinus, two-pole His bundle, and right ventricular/atrial (RV/A) electrodes were placed. Ventricular S1–S1 450-ms pacing showed auriculoventricular dissociation, ruling out left and right bypass. Atrial S1–S2 stimulation at 500/270 ms A‑H for 88 ms did not induce supraventricular tachycardia. Repeated S1–S2 and S1–S2–S3 stimulations along with an intravenous infusion of isoproterenol also did not induce supraventricular tachycardia; however, frequent premature beats were noted between two sinus beats. The sinus rhythm was not readjusted, which was similar to findings in patients with interpolated premature beats. The H wave was present in front of the V wave during the premature beats, and no A wave was noted. The H‑V interval was equal to the H‑V interval of the previous sinus rhythm, lasting 77 ms (Figs. 3 and 4).

The A‑H conduction interval was 95 and 543 ms via the fast pathway and slow pathway, respectively, during sinus rhythm.

Together, these ESP findings confirmed that sinus rhythm excitation was transmitted to the ventricle from both the fast and slow pathways, and the patient was therefore diagnosed with dual AV nodal pathways and double ventricular response.

Radiofrequency ablation of the slow pathway was performed to improve AV conduction. The power of the discharge was 26–30 W at a temperature of 48–55 °C. Nodal escape was noted, and after 160 s of discharge, the premature beats disappeared. The EPS was repeated and there was no evidence of dual AV nodal pathways and no tachycardia occurred. There were no premature beats and so-called accelerated idioventricular rhythm present on postoperative ECG monitoring and 24-h Holter ECG. The patient was followed up for 6 months: He did not experience any additional symptoms, and the ECG and 24-h Holter ECG were normal.

## Discussion

When a dual AV nodal pathway is present, a single supraventricular excitation is simultaneously transmitted from both the fast and slow pathways to the ventricles, causing the ventricles to be excited twice. This is an interesting electrophysiological phenomenon known as a ventricular double response. This phenomenon was first reported by Csapo et al. in 1979, and the common manifestation is irregular, nonreentrant supraventricular tachycardia [1, 2]. This electrophysiological phenomenon occurs when: (1) unidirectional retrograde block is present in both pathways; and (2) a slow pathway conduction delay ensures a sufficient A‑H interval, enabling the supraventricular excitation to transmit via the fast pathway and the slow pathway at the same time, thereby causing the second ventricular excitation. Clementy et al. reported one case of sinus excitation-induced ventricular double response that led to tachycardia-induced cardiomyopathy [3].

The case described in this report is unique in that the double ventricular response manifested as interpolated premature beats on the ECG, the majority of which occurred in a trigeminal pattern. Although no other case report or case series have described a similar finding, this case highlights the importance of EPS considering that the QRS configuration on the ECG was similar to that of sinus excitation and this patient’s condition was mistaken for junctional or ventricular (high position) premature contractions. Instead, EPS detected the H‑wave before the V‑wave during the premature beats, and the H‑V interval of premature beat was equal to the H‑V interval measured during sinus rhythm. This EPS finding is different from the characteristic finding of ventricular premature contractions originating from the His bundle region or junctional premature contractions. Therefore, this patient was diagnosed with dual AV nodal pathways and double ventricular response.

**Fig. 5:**
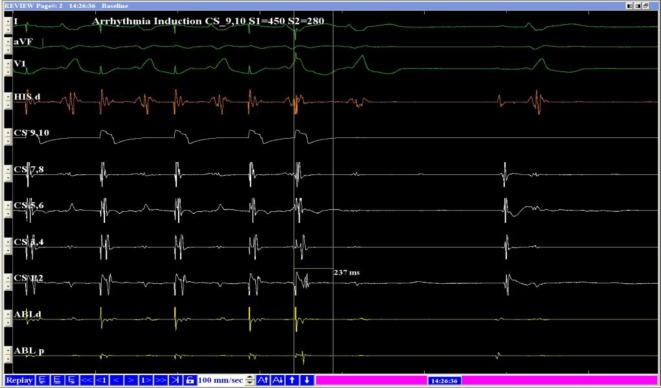
Atrial stimulation, S1–S2 450/280 ms. Conduction via fast pathway, AH = 247 ms

**Fig. 6:**
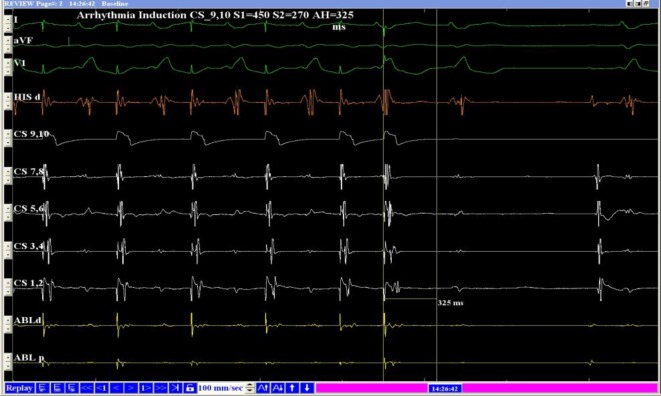
Atrial stimulation, S1–S2 450/270 ms. Conduction via slow pathway; conduction via fast pathway, AH = 325 ms; AH jump = 88 ms

The A‑H intervals measured during the fast and slow pathways were 95 and 543 ms, respectively. When the atrial S1–S2 protocol of stimulation was used, the A‑H jump was 88 ms. The A‑H interval was 325 ms (Figs. 5 and 6), which was shorter than the A‑H interval during double ventricular responses in the sinus rhythm when the excitation is transmitted via the slow pathway. A possible explanation for this finding was that slow pathway conduction was delayed during the double ventricular response. Slow-pathway radiofrequency ablation was effective, and these so-called premature beats disappeared. Holter ECG confirmed the disappearance of the premature beats, further supporting the authors’ diagnosis.

In this case, junctional or ventricular (high position) premature contractions could not be identified based on the configuration of the premature beats on the surface ECG. This likely led to the misdiagnosis of interpolated premature ventricular contractions, explaining why the previously prescribed medical therapy was ineffective. The Holter ECG also provided several clues that led to the diagnosis of double ventricular response and dual AV node pathways. For example, the Holter ECG showed a group of so-called spontaneous accelerated ventricular rhythms. These rhythms were compared with the ECG map of trigeminy during the premature beats. It was noted that the QRS wave configurations were consistent with those generated by the premature beats. In fact, this was because the sinus P‑wave was transmitted via the slow pathway, causing the P‑wave to fall on the T‑wave or the terminal QRS wave (Fig. 2). The authors therefore considered the possibility of dual AV nodal pathways and double ventricular responses before the EPS and catheter ablation. The patient experienced a similar rhythm during the EPS. Because the A–H interval during accelerated idioventricular rhythm was the same as the A–H interval measured during the double ventricular responses during the sinus rhythm when the excitation was transmitted via the slow pathway (Fig. 4), the presence of the double ventricular responses was confirmed.

In conclusion, if the authors had not considered the possibility of this electrophysiological phenomenon before carrying out radiofrequency ablation of the slow pathway, it would have been easy to mistakenly search the His bundle region for a discharge that induces ventricular premature contractions or even conduct blind ablation beside the His bundle, which may have led to His bundle injury. Therefore, improving a physician’s ability to analyze surface ECGs in order to identify rare electrophysiological phenomena may greatly reduce operative times for arrhythmia-related procedures and prevent serious complications caused by blind ablation due to misdiagnosis.
